# Is the surgical site infection rate higher in sublay or onlay mesh repair of incisional hernia?

**DOI:** 10.1016/j.amsu.2021.01.028

**Published:** 2021-01-21

**Authors:** Rashid Ibrahim, Sabry Abounozha, Talal Alshehri

**Affiliations:** a University Hospitals Plymouth NHS Trust, Plymouth, UK; b Northumbria Healthcare NHS Foundation Trust, Northumbria, UK; c Imam Abdulrahman Alfaisal Hospital, Riyadh, Saudi Arabia

**Keywords:** Incisional hernia, Onlay, Surgical site infection, Sublay, Wound infection

## Abstract

A best evidence topic has been constructed using a described protocol. The three-part question addressed was: In mesh repair of incisional hernia, which technique has a lower rate of surgical site infection (SSI), Sublay or Onlay? The best evidence showed that there is no statistically significant difference in the rate of SSI among the two techniques.

## Highlights

• IMesh repair of incisional hernia.
• Surgical site infection (SSI).
• Onlay vs Sublay.

## 1 Introduction

This BET was designed using a framework outlined by the International Journal of Surgery [1]. This format was used because a preliminary literature search suggested that the available evidence is of insufficient quality to perform a meaningful meta-analysis. A BET provides evidence-based answers to common clinical questions, using a systematic approach of reviewing the literature.

## 2 Clinical scenario

You are consenting a 56 year old male with incisional hernia, for open mesh repair. The patient is known diabetic and he is on immunosuppressant for a previous renal transplant, he is quite concerns about post-operative wound infection.

## 3 Three-part question

[In open mesh repair of incisional hernia] [which techniques has lower SSI rate] [Sublay or Onlay]?

## 4 Search strategy

### 4.1 Embase 1974 to October 2020 using the OVID interface

[Incisional hernia] AND [mesh ] AND [repair OR repairs ] AND [onlay] AND [sublay]AND [surgical site infection OR SSI OR wound infection OR infection]

### 4.2 Medline using the PubMed interface

[Incisional hernia] AND [mesh] AND [repair OR repairs] AND [onlay] AND [sublay]AND [surgical site infection OR SSI OR wound infection OR infection] The results were limited to English articles and human studies.

## 5 Search outcome

A total of 54 articles were identified after the removal of duplicates. Of these 40 were excluded on the basis of title and abstract. After full-text assessment of 14 articles another 8 articles were excluded because they did not include the information needed to compare the two techniques. A total of 6 articles (3 randomized controlled trials, one prospective and 2 retrospective studies) were identified to provide the best evidence to answer the question.

## 6 Result

See the table.Table. 1

**Table. 1:** Literature search result.

Author, date of publication, journal and country	Study type and level of evidence	Patient group + Follow up	Outcomes	Key results		Additional comments
Sevinç et al. 2018 Turk J Surg Turkey	Randomized controlled trial level II	Total of 100 with incisional hernia **Group 1**: 50 sublay **Group2**: 50 onlay median follow-up was 37.1 (26.6–46.5) months	Primary endpoint: Incidence of SSI	(ER) rate was: Group1 = 2 (4%) Group2 = 2 (4%) P = 1 Difference is not statistically significant		-Single centre, -Small sample size, -Short period of follow up -Nothing mentioned about the methods of diagnosis of SSI
Venclauskas et al. 2010 Hernia Lithuania	Randomized controlled trial level II	Total 107 patients underwent mesh repair Group1: 57onlay Group2: 50 sublay median follow-up 12 months	Primary endpoint: Incidence of SSI	Group1: 8 (14%) Group2: 1 (2%) (P = 0.025). Difference is statistically significant		Single centre, -Small sample size, -Short period of follow up -Nothing mentioned about the methods of diagnosis of SSI
Manzoor Ahmed1 et al. 2019 J Coll Physicians Surg Pak Pakistan	Multicenter, Randomized, Controlled Trial levelII	Total 65 patients underwent mesh repair Group1: 33onlay Group2: 32 sublay median follow-up Six months.	Primary endpoint: Incidence of SSI	Group1: 6 (9.23%) Group2: 3 (4.61%) (P = 0.304). Difference is not statistically significant		-Small sample size, -Short period of follow up -Nothing mentioned about the methods of diagnosis of SSI
Kumar et al. 2012 Indian J Surg India	Prospective study level III	Total 63 patients Randomized into: Group 1: 45 onlay Group 2: 18 sublay follow-up 60 months	Primary endpoint: Incidence of SSI	Group 1 = 6 (13.33%) Group 2 = 2 (11.11%) Difference is not statistically significant	-Single centre, -Small sample size -No randomization - sample size is not equal between 2 groups -Nothing mentioned about the methods of diagnosis of SSI	
John J. Gleysteen, Arch Surg. 2009 UK	Retrospective study level III	A total of 125 patients Group 1: 75 onlay Group 2: 50 sublay Follow-up averaged 64 months	Primary endpoint: Incidence of SSI	Group1 = 9 (12.0%) Group2 = 2 (4.0) (P = 0.12) Difference is not statistically significant	-Single centre, -small sample size, -Retrospective -Nothing mentioned about the methods of diagnosis of SSI	
Nadia Saeed et al. JPMI (2015) Pakistan	Retrospective cohort study, level III	80 patients underwent Mesh repair of incisional hernia Group 1: 40 onlay Group 2: 40 sublay No follow up mentioned	Primary endpoint: Incidence of SSI	Group 1 = 2 (5%) Group 2 = 4 (10%) (P = 0.019) Statically significant	-Single centre, -small sample size, -Retrospective -Nothing mentioned about the methods of diagnosis of SSI	

## 7 Discussion

Repair of incisional hernia is regarded as one of the most challenging general surgical procedure, due to the high recurrence rate and post-operative morbidity [2]. Open mesh repair (onlay and sublay technique) is proved to be superior to suture repair [3]. However due to the presence of mesh this technique is not without morbidity such as wound complication like seroma formation and infection [4]. In this article, we have reviewed the best evidences which compare the rate of surgical site infection among Onlay and Sublay techniques.

In 2012, Venclauskas et al. [5] conducted a randomized control trial to evaluate the rate of SSI among onlay and sublay group, their conclusion was that sublay technique has statistically significant lower rate of SSI compared to Onlay technique. In contrast to these findings, Saeed et al. [6] in 2014 published a retrospective study showing that the incidence of wound infection is actually significantly higher among the Sublay rather than the Onlay group.

However, despite these contradicting results, four studies in our review including two randomised controlled trials showed no statistically significant difference in SSI rate between Only and Sublay mesh repair these studies were conducted by Sevinç et al., Manzoor et al., Kumar et al. and Gleysteen [[7], [8], [9], [10]].

### 7.1 Clinical bottom line

The best evidence showed no statistically significant difference in SSI rate between only and Sublay mesh repair of incisional hernia.

### 7.2 Limitation of this review

1 Small sample size in most articles
2. Shorter period of follow in most articles.
3. Nothing mentioned in all articles about the methods of diagnosis of SSI.

## Ethical approval

Not applicable.

## Sources of funding

None.

## Author contribution

RI: conducted the literature search and wrote the paper.

SA: assisted in the literature search and Writing of paper.

TA: assisted in writing of paper.

## Consent

Not applicable.

## Registration of research studies

In accordance with the Declaration of Helsinki 2013, all research involving human participants has to be registered in a publicly accessible database. Please enter the name of the registry and the unique identifying number (UIN) of your study.

You can register any type of research at http://www.researchregistry.com to obtain your UIN if you have not already registered. This is mandatory for human studies only. Trials and certain observational research can also be registered elsewhere such as: ClinicalTrials.gov or ISRCTN or numerous other registries.

## Guarantor

Rashid Ibrahim (RI)1*, Sabry Abounozha (SA)2.

## Declaration of competing interest

None.
